# Supplementary material for: TAL effector driven induction of a SWEET gene confers susceptibility to bacterial blight of cotton
Source: Nat Commun. 2017 May 24;8:15588. doi: 10.1038/ncomms15588 (PMC5458083; doi:10.1038/ncomms15588)
Supplement: Supplementary Information — Supplementary Figures and Supplementary Tables. [file ncomms15588-s1.pdf]

Avrb6

5’-MDPIRSRTPSPARELLPGPQPDGVQPTADRGVSPPAGGPLDGLPARRTMSRTRLPSPPAPSPAFSAGSFS

DLLRQFDPSLFNTSLFDSLPPFGAHHTEAATGEWDEVQSGLRAADAPPPTMRVAVTAARPPRAKPAPRRR

AAQPSDASPAAQVDLRTLGYSQQQQEKIKPKVRSTVAQHHEALVGHGFTHAHIVALSQHPAALGTVAVKY

QDMIAALPEATHEAIVGVGKQWSGARALEALLTVAGELRGPPLQLDTGQLLKIAKRGGVTAVEAVHAWRN

ALTGAPLNLTPEQVVAIASHDGGKQALETVQRLLPVLCQAHGLTPEQVVAIASNIGGKQALETVQALLPV

LCQAHGLTPEQVVAIASNGGGKQALETVQRLLPVLCQAHGLTPEQVVAIASHDGGKQALETVQRLLPVLC

QAHGLTPEQVVAIASHDGGKQALETVQRLLPVLCQAHGLTPEQVVAIASNIGGKQALETVQRLLPVLCQA

HGLPPEQVVAIASHDGGKQALETVQRLLPVLCQAHGLTLDQVVAIASNIGGKQALETVQRLLPVLCQAHG

LTPQQVVAIASNSGGKQALETVQRLLPVLCQAHGLTPEQVVAIASHDGGKQALETVQRLLPVLCQAHGLT

PEQVVAIASHDGGKQALETVQRLLPVLCQAHGLTPEQVVAIASHDGGKQALETVQRLLPVLCQAHGLTPA

QVVAIASNNGGKQALETVQRLLPVLCQAHGLTPEQVVAIASNGGGRPALESIVAQLSRPDPALAALTNDH

LVALACLGGRPALDAVKKGLPHAPALIKRTNRRIPERTSHRVADHAQVVRVLGFFQCHSHPAQAFDDAMT

QFGMSRHGLVQLFRRVGVTELEARSGTLPPASQRWDRILQASGMKRAKPSPTSTQTPDQASLHAFADSLE

RDLDAPSPMHEGDQTRASSRKRSRSDRAVTGPSAQQSFEVRVPEQRDALHLPLSWRVKRPRTSIGGGLPD

PGTPTAADLAASSTVMREQDEDPFAGAADDFPAFNEEELAWLMELLPQ-3’

Supplementary Figure 1. Avrb6 amino acid sequence. The first amino acid of each TAL effector repeat is highlighted in blue. The RVD of each repeat is underlined.

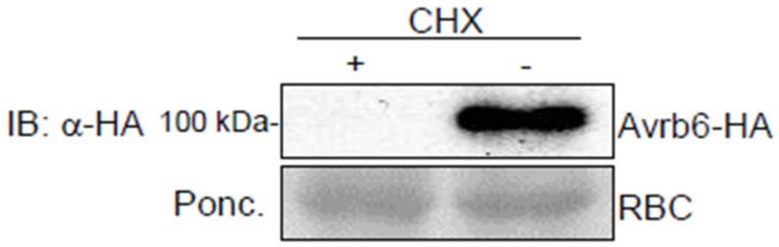

Supplementary Figure 2. Cycloheximide (CHX) suppresses Avrb6 protein synthesis in a cotton transient assay. Cotyledons from two-week-old Ac44E cotton were infiltrated with *Agrobacterium* carrying *35S::avrb6*-HA at OD_{600} = 0.6 with or without 50μM CHX. Tissues were harvested 4 dpi and subjected to immunoblotting with α-HA antibody (top panel). Ponceau S staining (Ponc) for RuBisCo (RBC) served as the protein loading control (bottom panel).

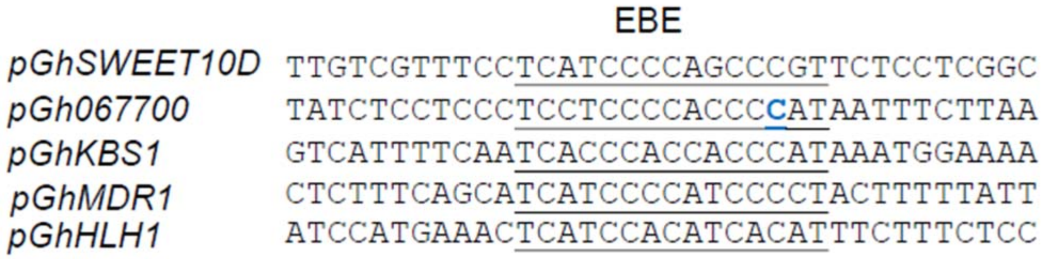

Supplementary Figure 3. Avrb6 EBEs of candidate genes. Avrb6 EBE of each candidate gene is underlined. The sequenced EBE of *Gh067700* bears a deleted nucleotide (indicated with the "C" highlighted in blue) when compared to the predicted Avrb6 EBE.

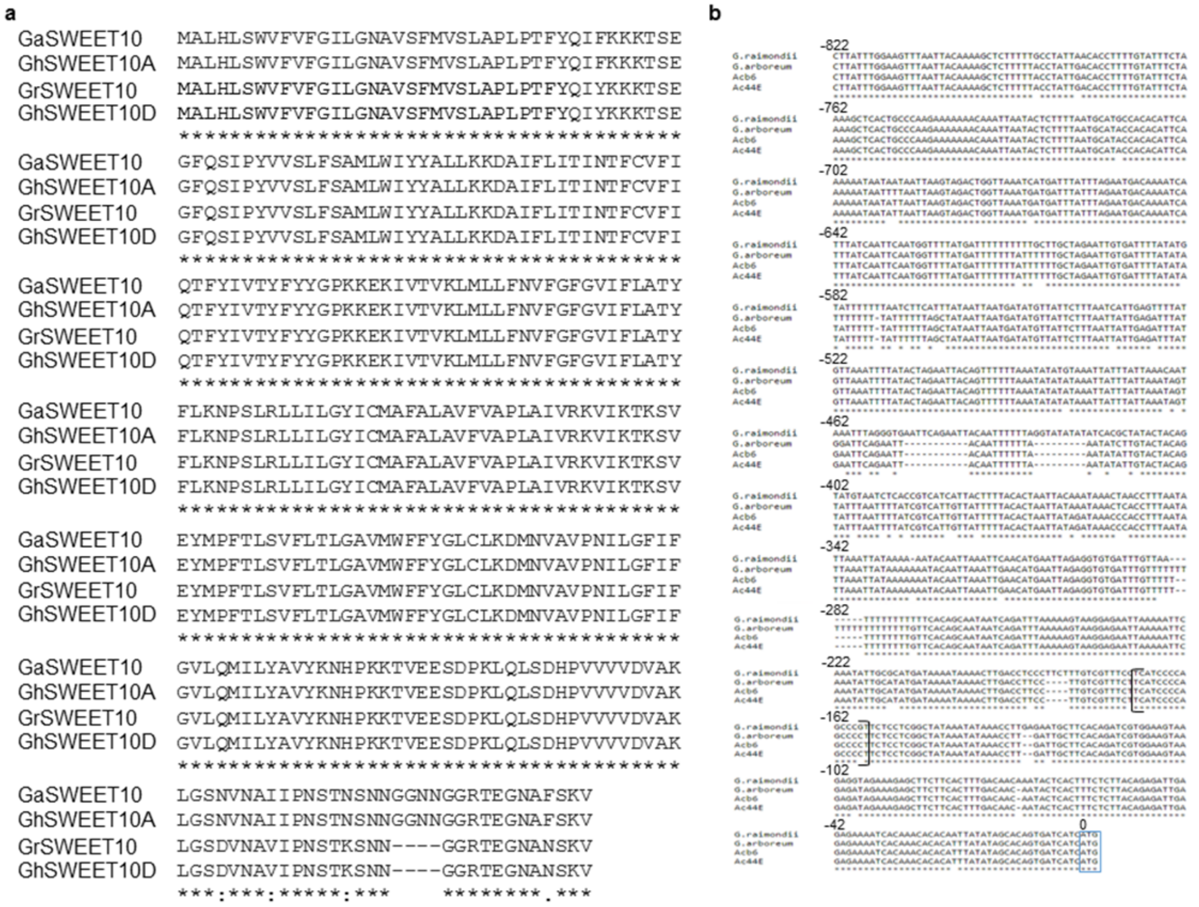

Supplementary Figure 4. Sequence alignment of SWEET10 coding sequences and promoters from *G. raimondii*, *G. arboreum*, and *G. hirsutum*. Alignment was performed with the protein coding sequences (A) and with the promoters using the Clustal Omega website ((http://www.ebi.ac.uk/Tools/msa/clustalo/) (B) with a hierarchical clustering approach. In (B), Avrb6 EBE is highlighted with a black bracket. ATG is highlighted with a blue box. The promoter sequences of *pGhSWEET10A* from A-subgenome in Ac44E and Acb6 are shown here.

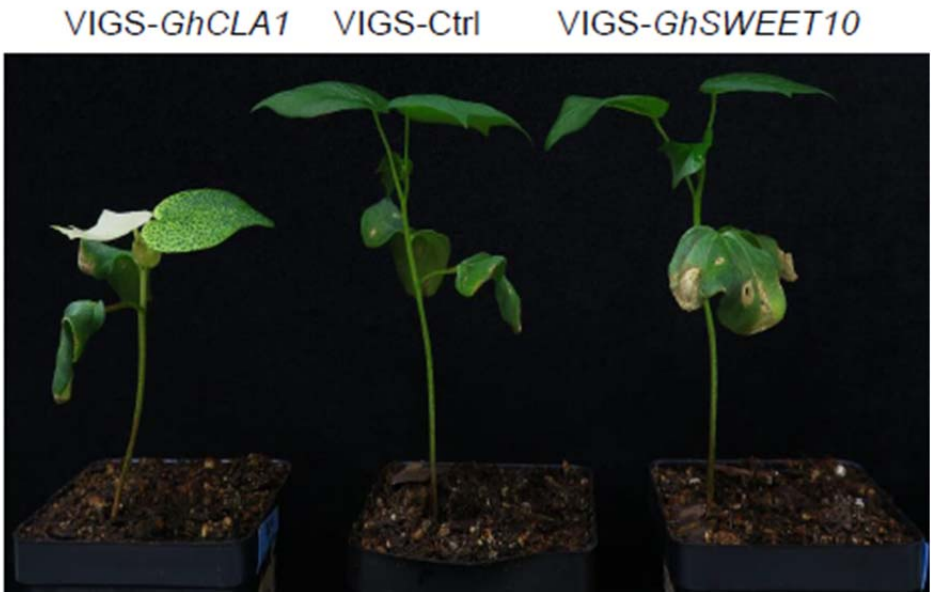

Supplementary Figure 5. VIGS-*GhSWEET10* did not affect cotton seedling growth. Pictures were taken at three weeks after inoculation. VIGS-*GhCLA1* is a visual marker for VIGS efficiency.

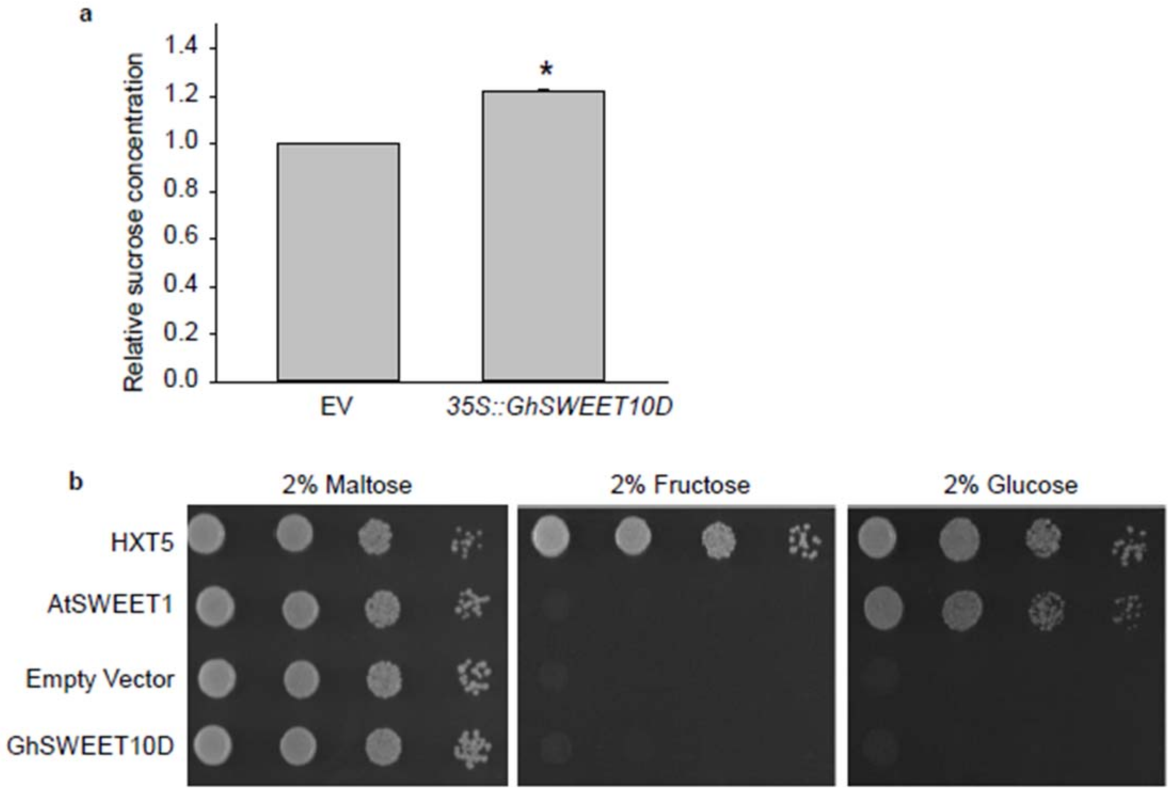

Supplementary Figure 6. GhSWEET10D sugar transport activity in *N. benthamiana* and yeast. (A) Expression of *GhSWEET10D* resulted in higher sucrose concentration in apoplasts of *N. benthamiana* leaves. *Agrobacterium* strain carrying *35S::GhSWEET10D* or an empty vector control (EV) was inoculated into *N. benthamiana* leaves. The sucrose content was normalized to a ratio relative to the EV. Error bar is SD and asterisk indicates significant difference using two-tailed t-test ($p<0.05$). The experiment was repeated twice. (B) GhSWEET10D does not complement yeast EBY4000 mutant, a hexose transport-deficient strain. The yeast was grown on the medium supplemented with 2% glucose or 2% fructose as the sole carbon source or 2% maltose as control. HXT5 from yeast and AtSWEET1 from Arabidopsis served as positive controls and empty vector served as a negative control.

Clade I

Gene ID	Gene Name
Gh_A02G0694	GhSWEET1aA
Gh_D02G0740	GhSWEET1aD
Gh_A02G1806	GhSWEET1bA
Gh_D03G1717	GhSWEET1bD
Gh_A11G3285	GhSWEET2aA
Gh_D11G2975	GhSWEET2aD
Gh_D10G0303	GhSWEET2bD
Gh_A11G2655	GhSWEET3A
Gh_D12G2692	GhSWEET3D

Clade II

Gene ID	Gene Name
Gh_A05G3128	GhSWEET4A
Gh_D04G0509	GhSWEET4D
Gh_A05G3127	GhSWEET5aA
Gh_D04G0510	GhSWEET5aD
Gh_A07G0535	GhSWEET5bA
Gh_D07G0604	GhSWEET5bD
Gh_A12G2152	GhSWEET6A
Gh_D12G2328	GhSWEET6D
Gh_A02G0950	GhSWEET7A
Gh_D03G0812	GhSWEET7D
Gh_A13G0907	GhSWEET8aA
Gh_D13G1148	GhSWEET8aD
Gh_A11G2446	GhSWEET8bA
Gh_D11G2763	GhSWEET8bD

Clade III

Gene ID	Gene Name
Gh_A08G0663	GhSWEET9aA
Gh_D08G2730	GhSWEET9aD
Gh_A07G0421	GhSWEET9bA
Gh_D07G0486	GhSWEET9bD
Gh_A12G1747	GhSWEET10A
Gh_D12G1898	GhSWEET10D
Gh_A03G0461	GhSWEET11A
Gh_D03G1078	GhSWEET11D
Gh_A07G0423	GhSWEET12aA
Gh_D07G0487	GhSWEET12aD
Gh_A07G0422	GhSWEET12bA
Gh_A11G0347	GhSWEET13A
Gh_D11G0404	GhSWEET13D
Gh_A04G0861	GhSWEET14aA
Gh_D04G1360	GhSWEET14aD
Gh_D02G1767	GhSWEET14bD
Gh_A01G0160	GhSWEET15A
Gh_D01G0202	GhSWEET15D

Clade IV

Gene ID	Gene Name
Gh_A11G2442	GhSWEET16aA
Gh_D11G2760	GhSWEET16aD
Gh_A13G1540	GhSWEET16bA
Gh_D13G1875	GhSWEET16bD
Gh_A10G1468	GhSWEET17aA
Gh_D10G1709	GhSWEET17aD
Gh_D02G0542	GhSWEET17bD
Gh_D13G1146	GhSWEET17cD
Gh_A13G1434	GhSWEET18A
Gh_D13G1763	GhSWEET18D

Supplementary Figure 7. Gene IDs and corresponding names of the cotton GhSWEET family. Tables are separated by different clades of GhSWEET family.

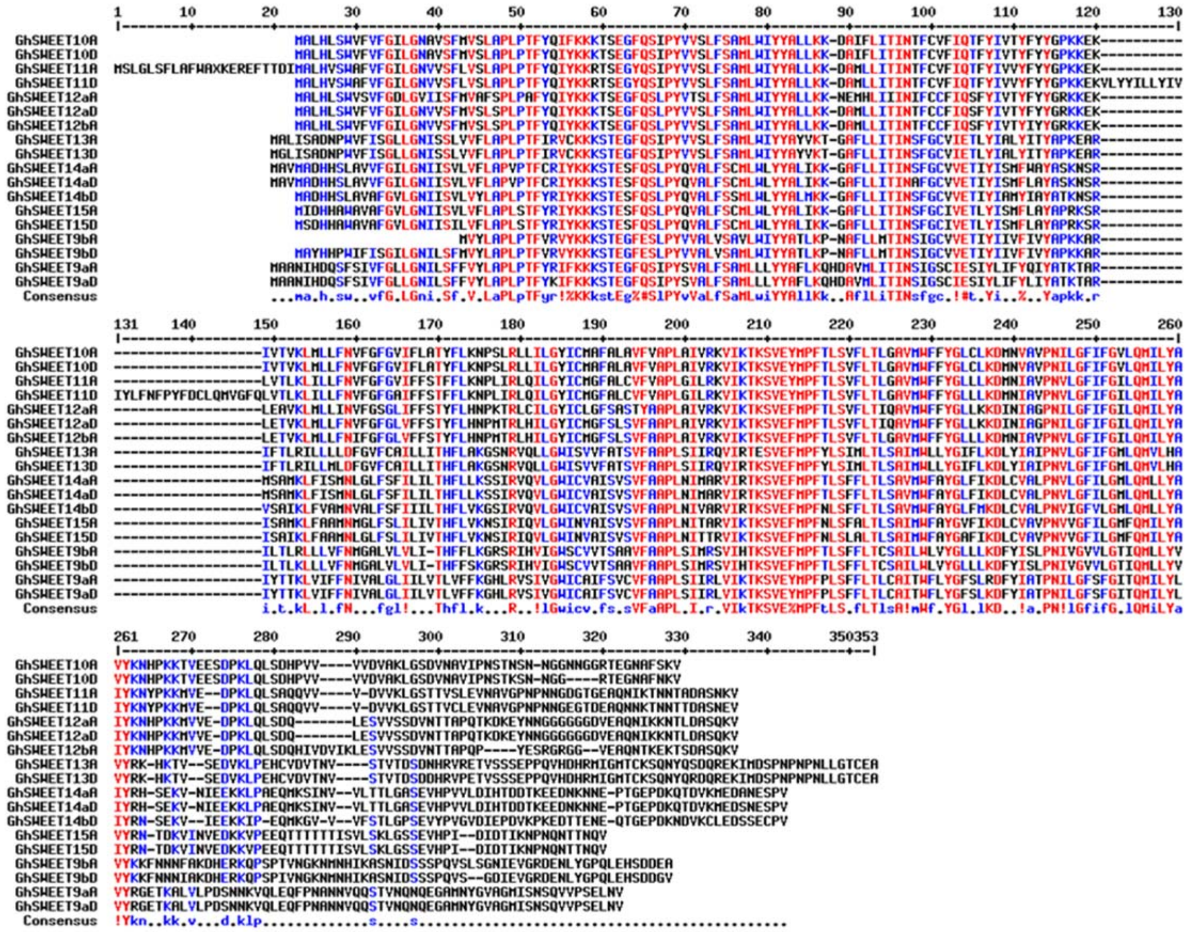

Supplementary Figure 8. Amino acid sequence alignment of cotton clade III GhSWEET proteins. Alignment was performed using the Multalin website (http://multalin.toulouse.inra.fr/multalin/) with a hierarchical clustering approach.

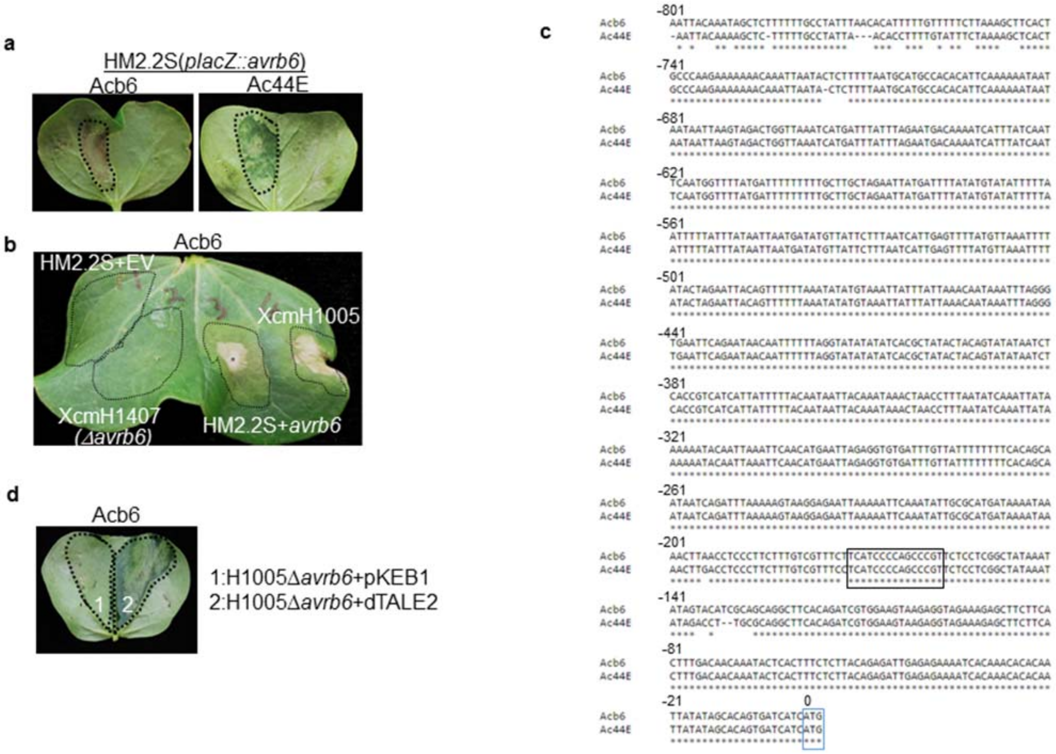

Supplementary Figure 9. Differential responses of Avrb6 in Ac44E and Acb6. (A) Avrb6 causes water-soaking on Ac44E, but HR on Acb6. Cotyledons of two-week-old plants were syringe-inoculated with *Xcm*HM2.2S (*placZ::avrb6*) at OD_{600} = 0.1. Pictures were taken at 4 dpi. (B) Strains carrying *avrb6*, XcmH1005 and HM2.2S+*avrb6* cause HR in Acb6. (C) Sequence alignment of the *GhSWEET10D* promoter in Ac44E and Acb6. Alignment was performed using the Clustal Omega website (http://www.ebi.ac.uk/Tools/msa/clustalo/) with a hierarchical clustering approach. Avrb6 EBE is highlighted with a black box. ATG is highlighted with a blue box. (D) dTALE2 causes water-soaking on Acb6. Cotyledons of two-week-old plants were syringe-inoculated with XcmH1005*Δavrb6* carrying dTALE2 or pKEB1 (empty vector) at OD_{600} = 0.1. Pictures were taken at 4 dpi.

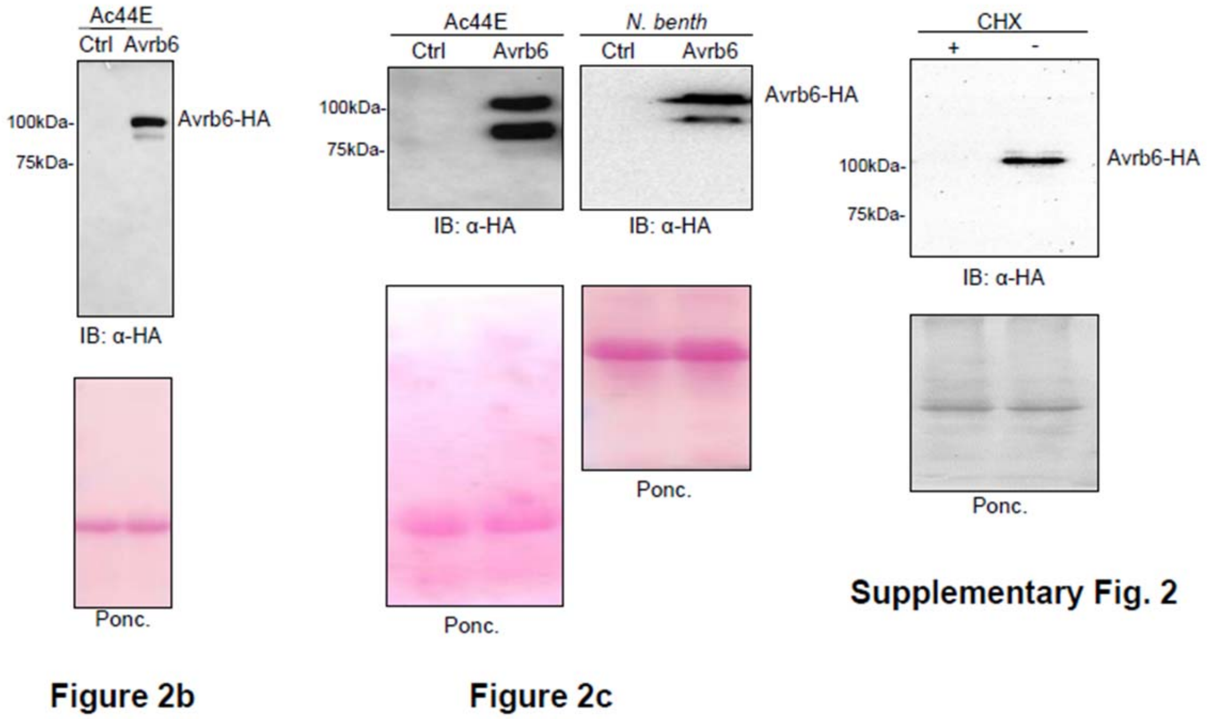

Supplementary Figure 10. Full blots of Figure 2b, 2c, and Supplementary Fig. 2.

Supplementary Table 1. Progressive MAUVE on pXcmH and pXcmN

pXcmH_left end	pXcmH_right end	pXcmN_left end	pXcmN_right end
79681	79764	9909	9992
-3939	-7800	10470	14331
-3463	-3935	14391	14877
16064	25400	16064	25399
25467	25611	25473	25616
59530	63360	35092	38921
7801	16063	0	0
25401	25466	0	0
25612	59529	0	0
63361	79680	0	0
0	0	9993	10469
0	0	14332	14390
0	0	14878	16063
0	0	25400	25472
0	0	25617	35091

Supplementary Table 2. RVD sequences and AnnoTALE names of XcmH1005 and XcmN1003 TAL effectors.

Annotated Name	Gene coordinates	RVD Sequence<br>1 2 3 4 5 6 7 8 9 10 11 12 13 14 15 16 17 18 19 20 21 22 23	AnnoTALE Name	Comments on coding sequence
**XcmH1005 chromosome**				
AvrB5	193705..196791	NI NS HD NI NS NS NS NS HD NI NS HD HD HD	TalDZ1 XcmH1005	
AvrB104	197283..200771	NI HD HD NS HD NG HD HD HD NI NI NI NN NI NG NG N* NG	TalDX1 XcmH1005	
PthN2	228643..231927	NI NG NI NI NS NI NN NI N* NN HD NN NS NG N* NN	TalEG1 XcmH1005	
AvrBn	2720814..2723696	NI NG NI HD HD HD HD NG HD HD NG NG	TalEE1 XcmH1005	
AvrB103	2748215..2751499	HD NI NG NI NI NI NG NG NS NG HD HD NI HD N* NN	TalEB2 XcmH1005	
$Tal6_{XcmH1005}$	4979648..4982734	NI NG NI NI NI NG HD HD NG NI HD NI NG NG	TalED1 XcmH1005	
**XcmH1005 plasmid (pXcmH)**				
AvrB4	8056..11649	NI NG NI NI NS NG NI HD NI NS NS HD HD HD NI NI HD HD NG	TalDY1 XcmH1005	

**Annotated Name**	**Gene coordinates**	**RVD Sequence**<br>**1 2 3 4 5 6 7 8 9 10**<br>**11 12 13 14 15 16 17 18 19**<br>**20 21 22 23**	**AnnoTALE Name**	**Comments on coding sequence**
Avrb6	12430..15516	HD NI NG HD HD NI HD NI NS HD HD HD NN NG	TalEC1 XcmH1005	
AvrB101	32633..36637	HD NG NS NG NI NI NS NG HD NS HD HD NS HD NG NI HD HD NG NS NG HD NG	TalEA1 XcmH1005	
AvrBIn	39817..43617	HD NI NG NI NI NI NG NG NS NG HD HD NI HD NS NG NS NG NG NG NG	TalEB1 XcmH1005	
AvrB102	50873..54367	NI NI NI NG HD NG NI HD NI HD NG NS NG HD NG HD NS NG	TalDW1 XcmH1005	
Avrb7	66226..69822	HD NI NG NI NI NI NS HD HD HD NS HD HD NS NS NG NS NG NG	TalEF1 XcmH1005	
**XcmN1003 chromosome**				
Tal1'$_{XcmN1003}$	194999..196450	HD	TalCP4 XcmN1003	Only a last, 57 bp (19 aa) repeat; IS element in 3' end
Tal2'$_{XcmN1003}$	2354470..2355501	HD	TalCP5 XcmN1003	Integrase insertion in 2nd repeat
AvrBn	2379939..2382821	NI NG NI HD HD HD HD NG HD HD NG NG	TalEE2 XcmN1003	
Tal4'$_{XcmN1003}$	2830732..2832598	NI HD HD NG HD NG NI NS NS HD	TalCC4 XcmN1003	Frameshift deletion at 11th and 12th repeats
Tal5'$_{XcmN1003}$	4988366..4990750	HD NG NI NI NG HD HD NG NI HD NI NG NG HD NI	TalEI1 XcmN1003	4 bp insertion in last repeat
**XcmN1003 plasmid (pXcmN)**				
PthN'	26436..29081	NI HD HD NI HD NI NG NI NN HD NI NG N* NN	TalEJ2 XcmN1003	Frameshift in 3' end
Tal7$_{XcmN1003}$	42258..45437	NI HD HD NS NG HD HD N* NN HD NN NS NG N* NN	TalEH1 XcmN1003	
PthN	51889..54969	NI HD HD NI HD NI NG NI NN HD NI NG N* NN	TalEJ1 XcmN1003	
PthN2	55295..58444	NI NG NI NI NS NI NN NI N* NN HD NN NS NG N* NN	TalEG2 XcmN1003	Repetitive element insertion in 3' end

Supplementary Table 3. *Xcm* strains and plasmids used in this study.

***Xcm* strains**

Strains	Relevant characteristics	Reference/Source
*Xcm*H1005	Spontaneous Rif^{r} derivative of *Xcm*H	Yang *et al.* 1994
*Xcm*N1003	Spc^{r}, Rif^{r} derivative of *Xcm*N	DeFeyter and Gabriel, 1991
*Xcm*H1407 (H1005Δ*avrb6*)	*avrb6*::Tn5-*gusA*, marker exchange mutant of *Xcm*H1005 (H1005Δ*avrb6*)	Yang *et al.* 1994
H1005 (*placZ::avrb6*)	*Xcm*H1005 with pUFR135	This study
HM2.2S	Mutant strain of H1005 lacking at least six *avr* genes	Chakrabarty *et al.* 1997
HM2.2S (vector)	HM2.2S with pUFR042	This study
HM2.2S (*avrb6*)	HM2.2S with pUFR127	Yang *et al.* 1996
HM2.2S (*placZ::avrb6*)	HM2.2S with pUFR135	This study
HM2.2S+pKEB1	HM2.2S with pKEB1	This study
HM2.2S+dTALE1	HM2.2S with dTALE targeting *GhSWEET10D* in pKEB1	This study
HM2.2S+dTALE2	HM2.2S with dTALE targeting *GhSWEET10* (A&D) in pKEB1	This study
HM2.2S+dTALE3	HM2.2S with dTALE targeting *GhMDR1* in pKEB1	This study
HM2.2S+dTALE4	HM2.2S with dTALE targeting *GhKBS1* in pKEB1	This study
*Xcm*H1005Δ*avrb6*+pKEB1	*Xcm*H1005Δ*avrb6* with pKEB1	This study
*Xcm*H1005Δ*avrb6*+dTALE1	*Xcm*H1005Δ*avrb6* with dTALE targeting *GhSWEET10D* in pKEB1	This study
*Xcm*H1005Δ*avrb6*+dTALE2	*Xcm*H1005Δ*avrb6* with dTALE targeting *GhSWEET10* (A&D) in pKEB1	This study
Xcm1	Field isolate from Plains, TX	This study
Xcm2	Field isolate from Plains, TX	This study
Xcm3	Field isolate from Plains, TX	This study
Xcm4	Field isolate from Plains, TX	This study
Xcm5	Field isolate from Plains, TX	This study
Xcm6	Field isolate from Plains, TX	This study
Xcm7	Field isolate from Matagorda, TX	This study
Xcm8	Field isolate from Lubbock, TX	This study
Xcm9	Field isolate from Seminole, TX	This study

Plasmids

Plasmid	Relevant characteristics	Reference/Source
pKEB1	Low copy, gateway-compatible pUFR047 derivative, Gm^r	This study
pRK2073	pRK2013 derivative, npt::Tn7,Kms, Sp^r, Tra$^+$, helper plasmid	Leong *et al.* 1982
pUFR042	IncW, Km^r, Gm^r, Mob$^+$, *lacZα*$^+$, Par$^+$	DeFeyter and Gabriel, 1991a
pUFR054	IncP, Tc^r, Mob$^+$, containing methylases *Xma*I and *Xma*II	DeFeyter and Gabriel, 1991b
pUFR127	5-kb fragment containing *avrb6* in pUFR042	DeFeyter and Gabriel, 1991a
pUFR135	2.9- + -0.5-kb *Bam*HI fragments from *Xcm*H containing intact *avrb6* in pUFR042; *lacZ*::*avrb6*	DeFeyter *et al.* 1993
pTRV-RNA1	pTRV encoding replicase, movement and cysteine-rich protein	Gao *et al.* 2011
pYL156-RNA2	TRV-based VIGS vector, Km^r	Gao *et al.* 2011

Supplementary Table 4. Primers used in this study.

Cloning and mutation primers

Gene	Forward Primer	Reverse Primer
Avrb6	GGACTAGT*ATG*GATCCCATTCGTTCGCG	GTCCCCCGGGCTGAGGCAATAGCTCCATC
PthN	GGACTAGT*ATG*GATCCCATTCGTTCGCG	GTCCCCCGGGCTGAGGCAATAGCTCCCTC
GhSWEET10D	CGGGATCC*ATG*GCTCTTCACTTGTCTTGGG	GAAGGCCTAACTTTGTTGAAAGCATTCCCTTC
pGhSWEET10D	CGGGATCCAAACCACATGGTGGGTGACA	CATGCCATGGCTTCCACGATCTGTGAAGCA
pGh067700	CGGGATCCAGAGGGGCTGACAGAGGCCCTA	CATGCCATGGTACGATCCGATCCCCAGTAAAAACAAG
pGhKBS1	CGGGATCCTTCCTTCCAAGAGATGACAAGAGC	CATGCCATGGAGAGATGTGAGCAGAGAGGGA
pGhMDR1	CGGGATCCTCGCTCCCAAAAACAATATAAATCA	CATGCCATGGGGACAGGACAGTGTTGTGGT
pGhHLH1	CGGGATCCGTCATTCCACATATCTCATCCCCA	CATGCCATGGAAGGATGATTGGATGGTTTGATAGA
pGhSWEET10D	CGTTTCCTCATCCCGGGCC	GAGGAGAACGGGCCCGGGATGAG

-mEBE	CGTTCTCCTC	GAAACG
VIGS-*GhSWEET10*	GGAATTCACGGCCCAAAGAAAGAAAAGAT	GGGGTACCAGGATGTTTGGAACGGCGAC
pGaSWEET10	CGGGATCCAAACCACATGGTGGGTGACA	CATGCCATGGCTTCCACGATCTGTGAAGCA

Note: The restriction enzyme sites are underlined and start codon is italicized. For point mutation primers, the mutation sites are underlined.

RT-PCR Primers

Gene	Forward Primer	Reverse Primer
GhSWEET10	ACGGCCCAAAGAAAGAAAAGAT	AGGATGTTTGGAACGGCGAC
Gh067700	TCACCAACCCTTTGCCTCAAT	AAGGTCTTCATCACCGCCAA
GhKBS1	TCCAGCGAAGATCCCCAGTA	GAACCCTTGACGTTTCGTGC
GhMDR1	CACCCTGGGTACTACATTGTCA	AGTGGCATTATCGCCCACAA
GhHLH1	ACCAACCAACCAAGAAACACC	CCCACCTGCTTTGTTGGAGT
GhACTIN	CCTCCGTCTAGACCTTGCTG	TCATTCGGTCAGCAATACCA
GaACTIN	CCTCCGTCTAGACCTTGCTG	TCATTCGGTCAGCAATACCA

qRT-PCR Primers

Gene	Forward Primer	Reverse Primer
GhSWEET9a	CAGCCAGGATATACACGAC	AAACTGAGAAGATGGCAC
GhSWEET10	GCCATTGTGAGAAAAGTC	TGAAGTACTCCAAAGATG
GhSWEET11	GCATTCTGGGAAACGTTG	ATCCAAAGCATCGCACTG
GhSWEET12a	GCTATCGTGAGAAAGGTC	ACCCTAGTATGTTTGGTC
GhSWEET12b	CCTTGCCAACATTTTATC	AGTAGCATAGCATCCTTC
GhSWEET14a	ACCAGTGCCAACATTCTG	ATCAACGCGTAATACAGC
GhSWEET14b	GGTGTCTTAGGTAACATC	AGCTGAACAATGCCACCTG
GhUBQ1	CTGAATCTTCGCTTTCACGTTATC	GGGATGCAAATCTTCGTGAAAAC
